# Supplementary material for: Physiological and molecular responses of lobe coral indicate nearshore adaptations to anthropogenic stressors
Source: Sci Rep. 2021 Feb 9;11:3423. doi: 10.1038/s41598-021-82569-7 (PMC7873073; doi:10.1038/s41598-021-82569-7)
Supplement: Supplementary file 1 — Supplementary Information 1. [file 41598_2021_82569_MOESM1_ESM.pdf]

Supplementary Files for:

Title: Physiological and molecular responses of lobe coral indicate nearshore adaptations to anthropogenic stressors

Authors:

- Kaho H Tisthammer , University of Hawaii at Manoa/San Francisco State University, kahot@hawaii.edu
- Emma Timmins-Schiffman, University of Washington, emmats@u.washington.edu
- Francois O Seneca, Scientific Centre of Monaco, fseneca@centrescientifique.mc
- Brook L Nunn, University of Washington, brookh@uw.edu
- Robert H Richmond University of Hawaii at Manoa, richmond@hawaii.edu

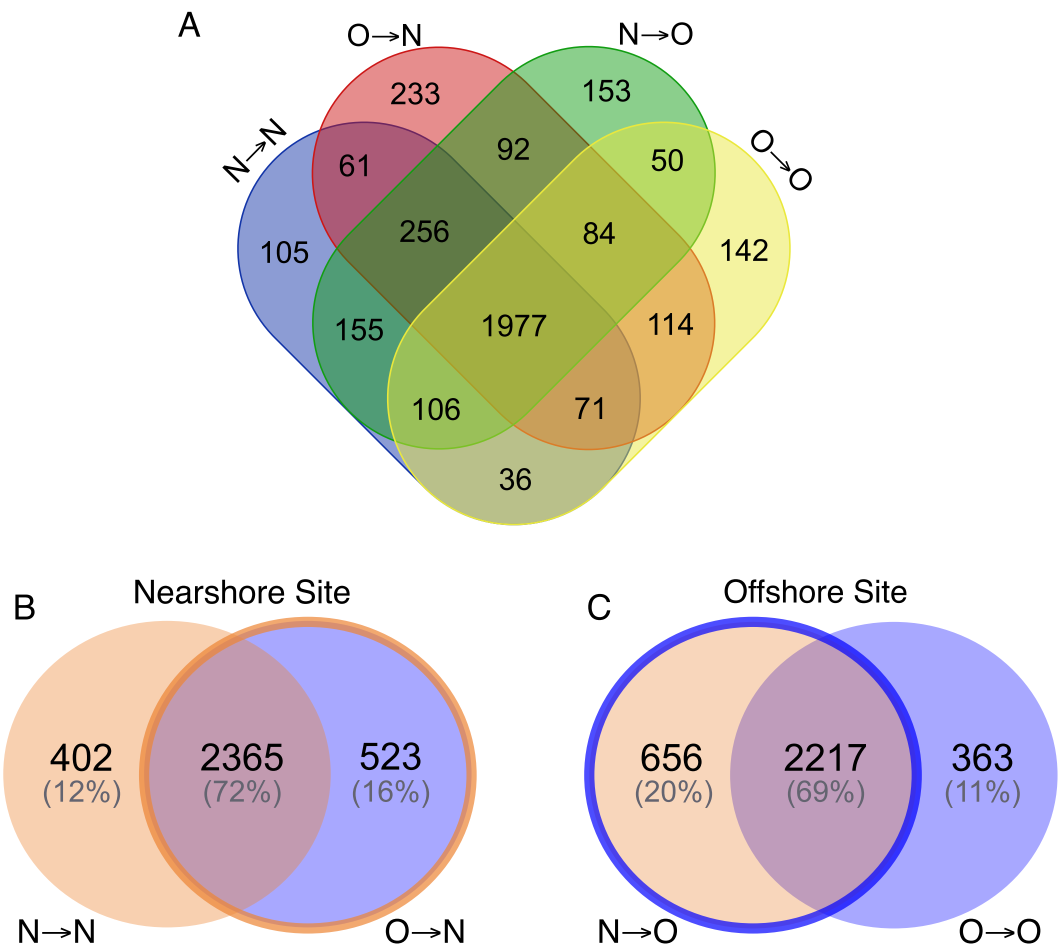

Figure S1. Venn diagrams showing the number of unique and overlapping proteins identified for each treatment. (A) All proteins identified for all four treatments, (B) The numbers of identified proteins at the nearshore site, and (C) the number of identified proteins at the offshore site.

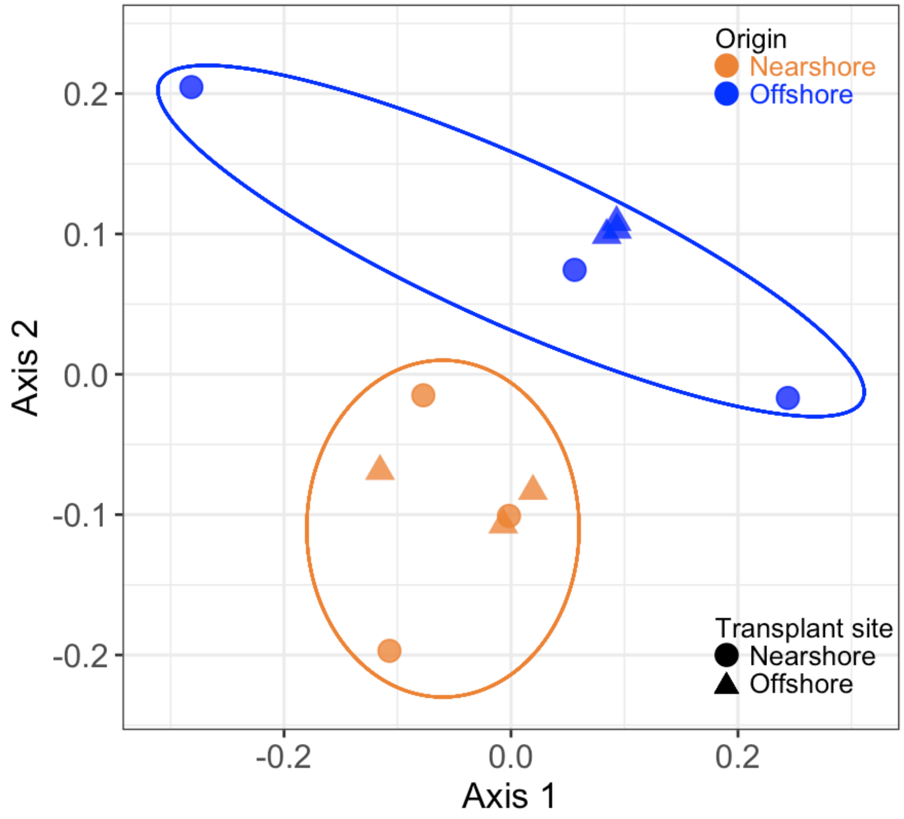

Figure S2. NMDS plot of protein abundances of the 12 coral samples used in LC-MS/MS. Nearshore corals (orange) and offshore corals (blue) showed a significant separation (R = 0.7074, P = 0.004), regardless of the transplant sites.

A

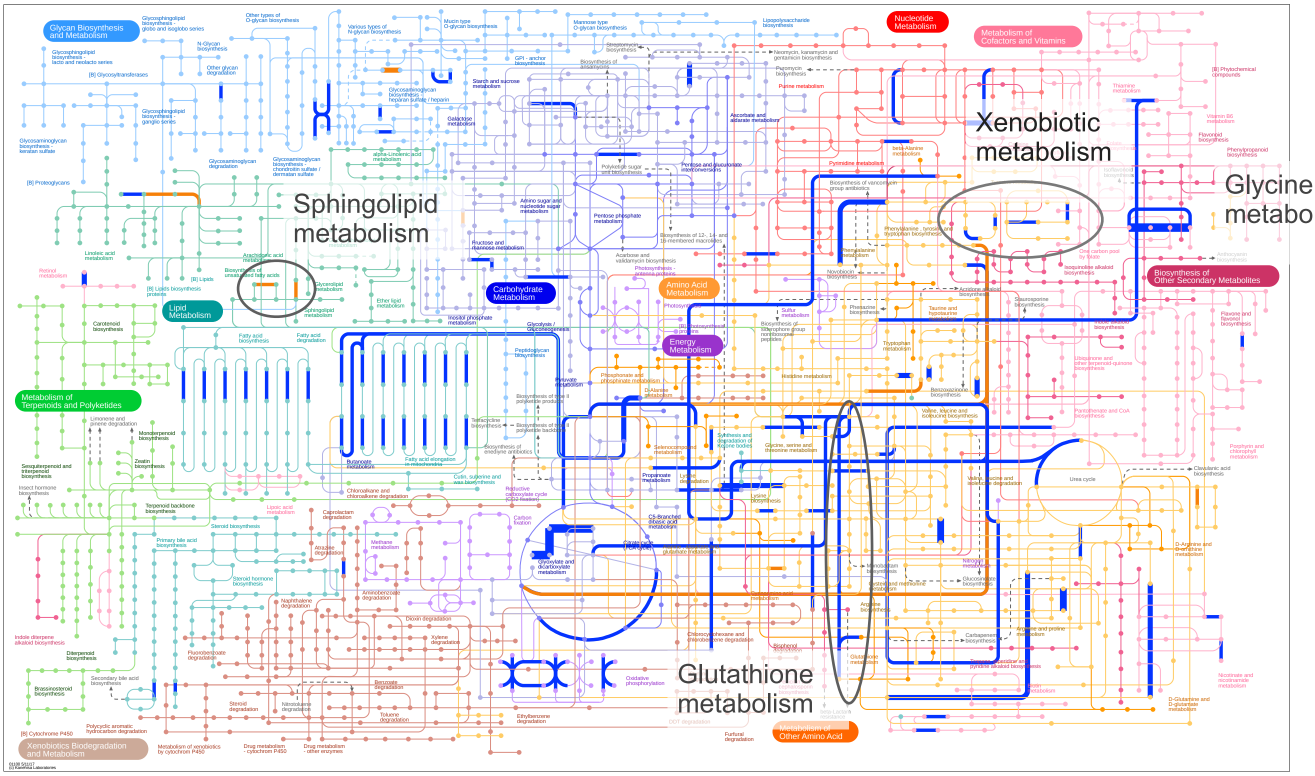

B

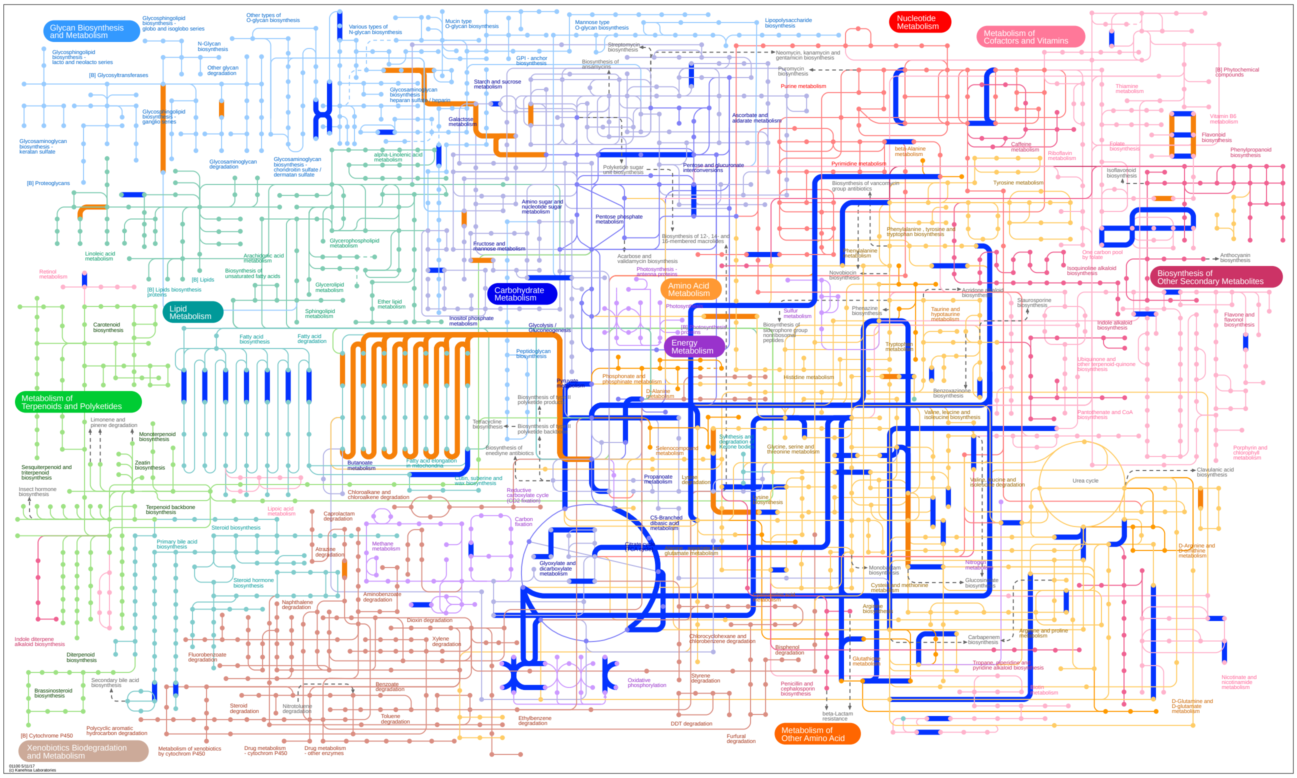

Figure S3. Metabolic pathway map generated by Interactive Pathways Explorer v.3 (iPath3[1], pathways.embl.de/) with significantly differentially expressed proteins of corals (A) at the nearshore site, and (B) at the offshore site. Blue lines indicate the pathways identified from significantly more abundant proteins in the offshore corals, and orange lines indicate those in the nearshore corals.

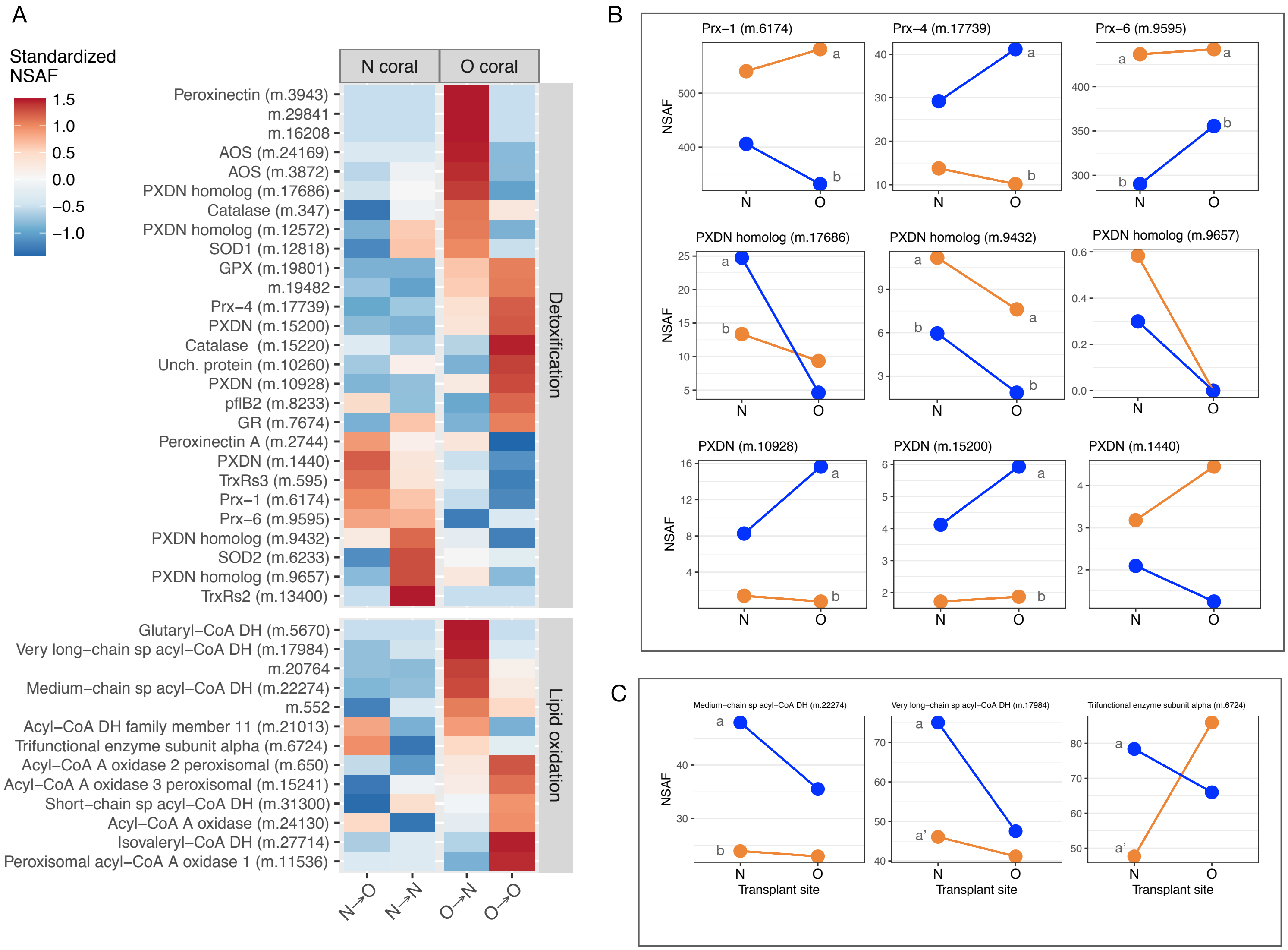

Figure S4. Relative abundance of proteins belonging to the GO terms detoxification (GO: 0098754) and lipid oxidation (GO:0034440). (A) A heatmap showing the relative abundance of each protein across the four treatments. The abundance (NSAF) of each protein was standardized across the four treatments (*i.e.* mean = 0, SD = ±1). (B) Individual protein abundances of detoxification proteins that showed contrasting responses between the populations (Peroxiredoxins and peroxidasin homologs). (C) Three lipid oxidation proteins that were significantly differentially abundant at the nearshore site (N-site). Letters in gray on (B) and (C) indicate significant difference in protein abundance between the populations at N-site or O-site (Qspec,| Z-stat|>2 and |Log2 fold change| > 0.5).

Table S1. Summary of significantly differentially expressed proteins between pairs of treatments. The numbers on the top represent the total number of proteins whose relative abundance was significantly different between the samples designated in comparison. The numbers in parentheses are for each treatment in the comparison (left *vs.* right).

Comparison	N→N *vs.* N→O	N→N *vs.* O→N	O→N *vs.* O→O	N→O *vs.* O→O	N→N *vs.* O→O
No. of differentially expressed proteins	135 (87, 48)	414 (138, 276)	440 (172, 268)	665 (155, 510)	644 (181, 463)
No. of unique proteins	573 (231, 342)	925 (402, 523)	976 (642, 334)	1019 (656, 363)	967 (577, 390)

Table S2. DNA sequence accession numbers for the *P. lobata* colonies used in the experiment

Source location	Sample ID	Marker	GenBank Accession No.
Nearshore	N1	H2	KY502354
Nearshore	N2	H2	KY502357
Nearshore	N3	H2	KY502358
Nearshore	N4	H2	KY502362
Nearshore	N5	H2	KY502364
Offshore	O1	H2	KY502366
Offshore	O2	H2	KY502370
Offshore	O3	H2	KY502369
Offshore	O4	H2	KY502368
Offshore	O5	H2	MF629151

Reference:

1. Darzi, Y., Letunic, I., Bork, P. & Yamada, T. iPath3.0: interactive pathways explorer v3. *Nucleic Acids Research* **46,** W510–W513 (2018).
